# Complement factor C5 inhibition reduces type 2 responses without affecting group 2 innate lymphoid cells in a house dust mite induced murine asthma model

**DOI:** 10.1186/s12931-019-1136-5

**Published:** 2019-07-24

**Authors:** Jack Yang, Ivan Ramirez Moral, Cornelis van ’t Veer, Alex F. de Vos, Regina de Beer, Joris J. T. H. Roelofs, B. Paul Morgan, Tom van der Poll

**Affiliations:** 1 Center of Experimental and Molecular Medicine, Amsterdam UMC, University of Amsterdam, Amsterdam, The Netherlands; 2 Department of Pathology, Amsterdam UMC, University of Amsterdam, Amsterdam, The Netherlands; 3 Division of Infectious Diseases, Amsterdam UMC, University of Amsterdam, Amsterdam, The Netherlands; 4 Division of Infection and Immunity, School of Medicine, Cardiff University, Cardiff, UK

**Keywords:** Complement system, Asthma, Allergic lung inflammation, House dust mite

## Abstract

**Background:**

Complement factor C5 can either aggravate or attenuate the T-helper type 2 (T_H_2) immune response and airway hyperresponsiveness (AHR) in murine models of allergic asthma. The effect of C5 during the effector phase of allergen-induced asthma is ill-defined.

**Objectives:**

We aimed to determine the effect of C5 blockade during the effector phase on the pulmonary T_H_2 response and AHR in a house dust mite (HDM) driven murine asthma model.

**Methods:**

BALB/c mice were sensitized and challenged repeatedly with HDM via the airways to induce allergic lung inflammation. Sensitized mice received twice weekly injections with a blocking anti-C5 or control antibody 24 h before the first challenge.

**Results:**

HDM challenge in sensitized mice resulted in elevated C5a levels in bronchoalveolar lavage fluid. Anti-C5 administered to sensitized mice prior to the first HDM challenge prevented this rise in C5a, but did not influence the influx of eosinophils or neutrophils. While anti-C5 did not impact the recruitment of CD4 T cells upon HDM challenge, it reduced the proportion of T_H_2 cells recruited to the airways, attenuated IL-4 release by regional lymph nodes restimulated with HDM ex vivo and mitigated the plasma IgE response. Anti-C5 did not affect innate lymphoid cell (ILC) proliferation or group 2 ILC (ILC2) differentiation. Anti-C5 attenuated HDM induced AHR in the absence of an effect on lung histopathology, mucus production or vascular leak.

**Conclusions:**

Generation of C5a during the effector phase of HDM induced allergic lung inflammation contributes to T_H_2 cell differentiation and AHR without impacting ILC2 cells.

**Electronic supplementary material:**

The online version of this article (10.1186/s12931-019-1136-5) contains supplementary material, which is available to authorized users.

## Background

Asthma is a heterogeneous disease characterized by airway hyperresponsiveness (AHR) and usually chronic airway inflammation dominated by a T-helper type 2 (T_H_2) response [1, 2]. The vast majority of allergic asthma patients are sensitized to house dust mite (HDM) and exposure to this abundantly present allergen causes respiratory symptoms such as coughing, wheezing and reversible airway obstruction [3].

The complement system is an important part of the innate immune system and consists of a network of proteins that when activated releases proteolytic fragments with pro-inflammatory properties. Activation of the complement system can occur through three pathways (i.e., the classical, lectin and alternative pathways), which lead to downstream proteolytic cleavage of C3 and C5, resulting in the release of the anaphylatoxins C3a and C5a [4, 5]. Recent investigations revealed a novel role for these anaphylatoxins and their receptors in the pathogenesis of asthma [6, 7]. In asthma patients, elevated levels of C3a and C5a were detected in the airways following allergen challenge [8]. While C3a signaling aggravates AHR [9] and drives allergic inflammation in different asthma models [10–12], C5a can exert both protective and detrimental effects during the course of an allergic inflammation. Prior to allergen sensitization, genetic deletion or pharmacological blockade of C5 or the C5a receptor (C5aR) resulted in a strongly enhanced allergic phenotype [13, 14]. Mechanistically, C5a/C5aR signaling regulated dendritic cells (DC) function, thereby favoring plasmacytoid DCs to suppress T-cell activation [13]. In addition, C5a/C5aR signaling in myeloid DCs hampered production of the chemokines CCL17 and CCL22, leading to an impaired recruitment of T_H_2 cells into the lung [15]. Furthermore, C5a/C5aR signaling can induce IL-12 production in antigen presenting cells and potentiates skewing toward T_H_1 responses [16]. In contrast, eliminating C5a/C5aR signaling after the sensitization phase reduced allergic lung inflammation [17] and AHR [18, 19]. The underlying mechanisms for this C5a mediated proallergic effect in an established inflammation environment is not well understood.

Group 2 Innate lymphoid cells (ILC2s) have been recognized to play an important role in type 2 immune responses [20, 21]. As an innate counterpart of T_H_2 cells, ILC2s orchestrate the allergic immune response by producing T_H_2 associated cytokines (IL-5 and IL-13) and presenting antigen to naïve T-cells for an effective T_H_2 cell development [22]. In the absence of T and B cells, the presence of ILC2s is sufficient to initiate and maintain an allergic lung inflammation and AHR in distinct mouse asthma models [23], emphasizing the significant contribution of ILC2s to the hallmarks of asthma. In earlier experimental asthma models blocking the C5a/C5aR axis, the type 2 response was primarily attributed to T_H_2 cells without assessing the contribution of ILC2s [13, 18]. We here studied the effect of C5 inhibition during the effector phase on the type 2 responses in the lung and AHR in a HDM induced asthma model.

## Material and methods

### Mice

Female BALB/c mice (8–12 weeks old) were purchased from Charles River (Maastricht, the Netherlands). Mice were housed under specific pathogen-free conditions receiving food and water ad libitum. All experiments were approved by the Animal Care and Use Committee of the Academic Medical Center.

### HDM asthma model

To induce allergic lung inflammation in mice, repeated HDM extract intranasal challenges were performed as described previously [24]. Briefly, mice were sensitized on day 0, 1, 2 and challenged on day 14, 15, 18, 19 with 25 μg HDM extract (Greer Laboratories, Lenoir, N.C., USA) or sterile saline. Prior to intranasal administration of HDM, all mice were anesthetized with isoflurane. BALB/c mice were injected intraperitoneally with a rat anti-mouse C5 monoclonal antibody (clone BB5.1; 1 mg/mouse) [25] or an irrelevant control antibody twice weekly (on days 13, 14, 17 and 18) during the challenge phase. Mice were euthanized 24 h after the last challenge. In all experiments citrate blood was collected from the vena cava inferior (4:1 v/v) and bronchoalveolar lavage (BAL) fluid was collected by airway lumen lavage with 2 × 0.5 ml PBS containing 10 mM EDTA, 10 mM benzamidine and 0.2 mg/ml soy bean trypsin inhibitor as described [24]. Cell counts were measured using a hemocytometer (Beckman Coulter, Fullerton, CA, USA) and cell differentiation was made by flow cytometric analysis. In one experiment the lavaged lungs were minced, followed by enzymatic digestion in RPMI medium with 5% Fetal Bovine Serum, 1% penicillin/streptomycin, liberase™ and DNAse at 37 °C for 30 min. Next, incubated cells were dissociated by aspiration through a 19-gauge needle to obtain single cells. Erythrocytes were lysed with sterile lysis buffer (Qiagen, Hilden, Germany). Unflushed lung, collected in a separate experiment, was used for pathology examination to avoid structural disruption as a consequence of BAL.

### Measurement of enhanced pause (PenH)

PenH was measured at day 19 by whole-body plethysmograph in conscious mice (Buxco Electronics, Troy, NY, USA) as described [24]. Mice were first subjected to aerosolized saline to determine nonspecific responsiveness, followed by increasing concentrations of aerosolized methacholine (3.1, 12.5, 25 and 50 mg/mL in saline for 3 min; Sigma-Aldrich). For each methacholine dose PenH values were measured over five minutes.

### Flow cytometry

Cells in BAL fluid were stained with CD3 FITC, CD11c PercP, Siglec F Alexa 647, CD11b PE-Cy7, viability dye APC Cy7 (all BD Biosciences, San Jose, CA, USA), Ly6G Alexa700 (Biolegend, San Diego, CA, USA), MHCII PE, and CD45 PE-eFluor610 (eBiosciences, San Diego, CA, USA) in the presence of Fc blocker (CD16/CD32, eBiosciences). Single cell suspensions from lungs were stained with CD4 FITC, CD45 PerCP-Cy5.5 (eBiosciences), GATA-3 Alexa 647, and viability dye APC-Cy7 (BD Biosciences). The following markers were used for the analysis of ILCs in lung tissue: Lineage (Lin) markers including CD3e, CD19, GR1, B220, Ter119, FcaR1 (all FITC, Biolegend), CD45 Alexa700, CD90 PE, ST2 Brilliant Violet 421 (Biolegend), CD49b PE-Cy7 (eBiosciences) and CD3 Percp-Cy5.5 (BD biosciences). Mediastinal lymph nodes (mLN) cells were stained with CD45 PerCP-Cy5.5, CD4 FITC, GATA-3 Alexa 647 (BD Biosciences) and IL-4 APC (Biolegend). For intracellular/intranuclear staining, cells were permeabilized and fixed using a FOXp3 Staining Buffer set (eBioscience) and subsequently stained with the appropriated markers. All appropriate Fluorescence Minus One (FMO) controls were used. Data were collected on a BD Biosciences Canto II flow cytometer or BD FACSAria™ III and analyzed using FlowJo software (Treestar, Palo Alto, CA, USA).

### Assays

C5a was measured in BAL fluid by ELISA. Purified rat anti-mouse C5a (clone I52–1486) was used as capture antibody, purified recombinant mouse C5a as standard and biotinylated rat anti-mouse C5a (clone I52–278) as detection antibody (all from BD Biosciences). Cytokines (IL-4, IL-5, IL-13), myeloperoxidase (MPO) and elastase were measured by ELISA (R&D systems, Minneapolis, MN, USA). Plasma total IgE was determined using rat-anti-mouse IgE as a capture antibody, purified mouse IgE as a standard, and biotinylated rat-anti-mouse IgE as detection (all from BD Biosciences) as described [24]. Plasma HDM-specific IgG1 was determined using HDM as capture and biotinylated rat-anti-mouse IgG1 as detection (BD Biosciences). BAL fluid IgM was determined as described [24], using rat-anti-mouse IgM as capture antibody, purified mouse IgM as standard and biotinylated goat-anti-mouse IgM (all from BD Biosciences) as detection. Total protein in BAL fluid was measured using Bio-Rad protein assay (Bio-Rad Laboratories, Veenendaal, Netherlands).

### Ex vivo stimulation of mediastinal lymph nodes (mLN)

Stimulations of mLN ex vivo were performed as described [24]. Briefly, mLN were harvested 24 h after the last challenge and filtered through 100 μm strainers. Single cells were seeded at a density of 2 × 10^5^ cells/well in 96-well round bottom plates (Greiner Bio-One, Alphen a/d Rijn, Netherlands) and incubated with 25 μg/ml HDM or PBS for four days at 37 °C with 5% CO. Supernatants were collected and stored at − 80 °C until analysis. In a separate experiment IL-4 production by CD4 T-cells in mLN were determined. To that end, mLN cells were stimulated with PMA (10 ng/ml) and ionomycin (1500 ng/ml; both Sigma-Aldrich) for five hours in the presence of Golgiplug (BD Biosciences) for the final three hours.

### Histology

Histological analysis was performed as described [24]. Briefly, after fixation in 10% formalin, four μm thick sections were stained with Hematoxylin and Eosin (H&E) to determine allergic inflammation properties such as edema, endothelialitis, peribronchial and perivascular inflammation and interstitial inflammation on a scale from 0 to 4 (0: absent; 1: mild; 2: moderate; 3: severe; 4: very severe). To examine mucus production, sections were stained with Periodic acid-Schiff (Pas-D) and scored for extent of goblet cells and mucous plugs on a scale from 0 to 3. Slides were coded and scored by a pathologist in a blinded fashion.

### Statistical analysis

Data were analyzed by Mann-Whitney U-test for comparison between groups. Two-way analysis of variance test followed by Tuckey’s multiple comparison test was used for groups of three or more. Experimental groups consisted of 6–8 mice. *P* ≤ 0.05 was considered statistically significant. All statistical analyses were performed using GraphPad Prism 7.

## Results

### C5 inhibition does not modify leukocyte influx in the airways upon HDM challenge

**Fig. 1:**
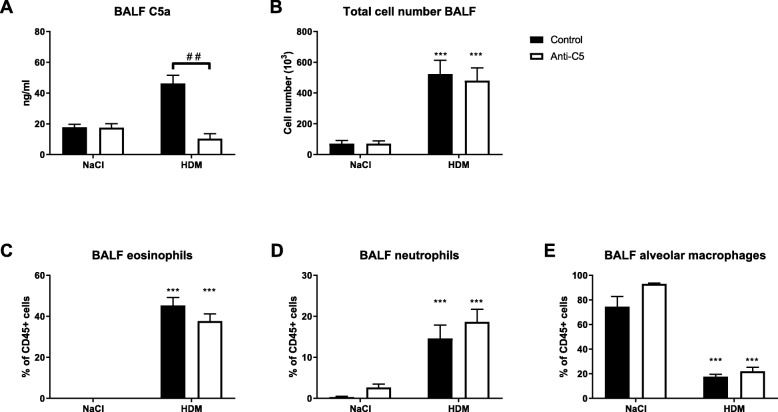
C5 inhibition does not modify leukocyte influx in the airways. Mice were injected intraperitoneally with anti-C5 or control antibody twice weekly during the challenge phase. (**a**) C5a concentration in bronchoalveolar lavage fluid (BALF) from saline (NaCl) or HDM challenged mice. (**b**) Total cell count in BALF. (**c**-**e**) Percentage of eosinophils, neutrophils and alveolar macrophages in BALF. Data are expressed as means ± SEM (*n* = 6–8 per group). *##P* < 0.01, ***P < 0.001 for comparison between NaCl and HDM within control or anti-C5 antibody injected mice

**Fig. 2:**
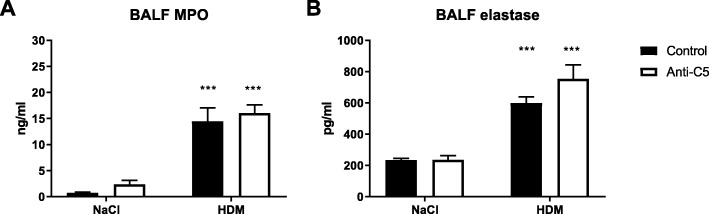
C5 inhibition does not alter neutrophil degranulation. (**a**) Myeloperoxidase and (**b**) elastase concentration in bronchoalveolar lavage fluid (BALF). All data are presented as means ± SEM (*n* = 6–8 per group). ***P < 0.001 for comparison between NaCl and HDM within control or anti-C5 mice

To investigate the contribution of C5 activation to allergic lung inflammation HDM sensitized mice were treated with a neutralizing anti-C5 mAb during repeated HDM challenges via the airways. HDM challenge resulted in increased C5a levels in BAL fluid. Treatment with anti-C5 mAb during the challenge phase reduced HDM-induced C5a concentrations to levels detected in unchallenged mice (Fig. 1a). Repeated HDM challenge triggered an influx of leukocytes into BAL fluid (Fig. 1b),which was the result of recruitment of eosinophils (Fig. 1c) and neutrophils (Fig. 1d). The number of alveolar macrophages in BAL fluid was lower in HDM challenged mice when compared with control animals (Fig. 1e). Anti-C5 did not modify leukocyte numbers or composition in BAL fluid. HDM challenge also caused neutrophil degranulation, as reflected by elevated concentrations of MPO and elastase in BAL fluid; this response was not altered by anti-C5 (Fig. 2a, b).

### C5 inhibition reduces T_H_2 but not ILC2 cell numbers following HDM challenge

**Fig. 3:**
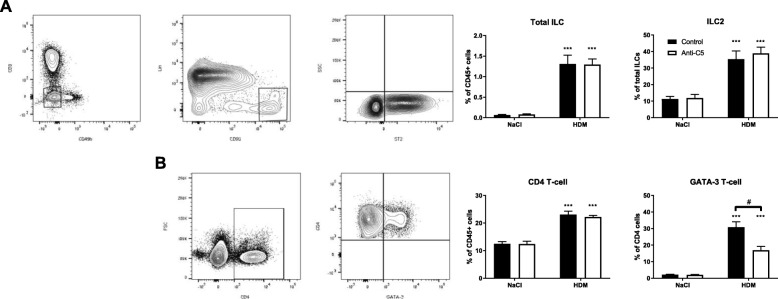
C5 inhibition reduces T_H_2 cells but not ILC2 following HDM challenge. Flow cytometry analysis of group 2 innate lymphoid cells (ILC2) and T-helper 2 cells (T_H_2) in lung tissue. All cells depicted here are pre-gated as single, viable and CD45^+^ cells. (A) Total ILCs were defined as CD45^+^Lin^−^CD3^−^CD49b^−^ CD90^+^ cells. Within the total ILC population ST2^+^ cells were regarded as ILC2 cells. (B) Total T-helper cells were defined as CD45^+^CD4^+^ cells. T_H_2 cells within total T-helper cell population stain positive for the transcription factor GATA-3. All data are means ± SEM (*n* = 8 per group). *#P* < 0.05 ***P < 0.001 for comparison between NaCl and HDM within control or anti-C5 mice

ILC2s and T_H_2 cells are both essential for the initiation and propagation of a type 2 immune response in allergic airway inflammation [22]. Therefore, we determined whether C5 inhibition influences the influx or expansion of these cells upon HDM challenge in sensitized mice. Total ILCs were defined as CD45^+^Lin^−^CD3^−^CD49b^−^CD90^+^ cells, and within the total ILC population, ILC2s were further identified by expression of ST2 (Additional file 1: Figure S1). C5 inhibition during the challenge phase did not affect the proportion of total ILC or ILC2s in the lungs (Fig. 3a). Likewise, the percentage of CD4 T-cells in the lungs was similar between the anti-C5 treated and control group following HDM challenge (Fig. 3b). However, the percentage of T_H_2 cells, defined as CD4^+^GATA-3^+^ cells, within the total CD4 T-cell population (Additional file 2: Figure S2) was significantly lower in anti-C5 treated mice.

### C5 inhibition reduces type 2 responses to HDM

**Fig. 4:**
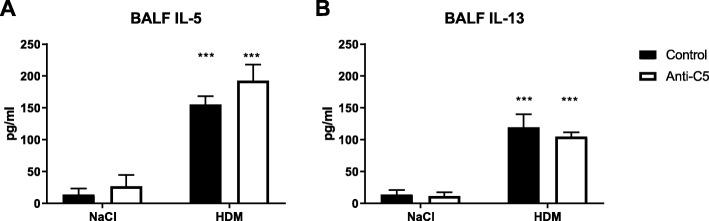
C5 inhibition does not modulate cytokine release in BALF following HDM challenge. (**a**, **b**) IL-5, IL-13 in bronchoalveolar lavage fluid (BALF) Data are depicted as means ± SEM (n = 6–8 per group). ***P < 0.001 for comparison between NaCl and HDM within control or anti-C5 mice

**Fig. 5:**
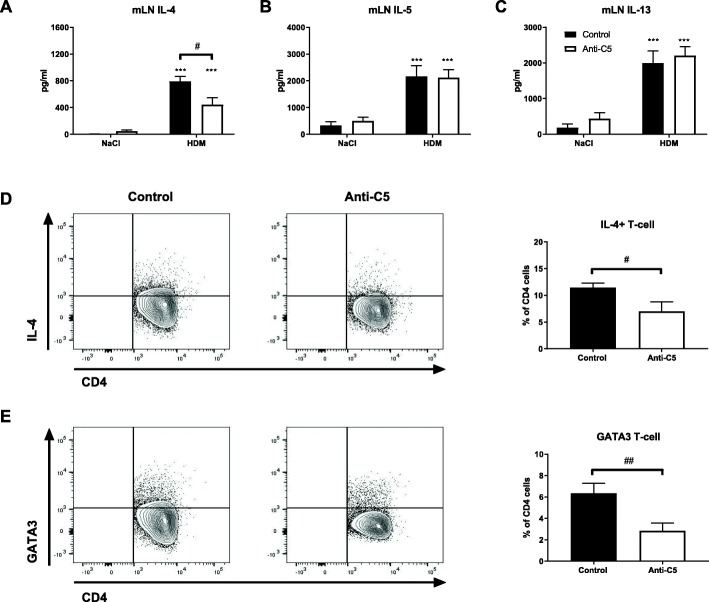
C5 inhibition mitigates IL-4 production and percentage of T_H_2 cell in the mLN following HDM challenge. Mediastinal lymph nodes (mLN) were harvested from HDM sensitized and challenged mice in both control and anti-C5 groups. In one experiment mLN cells were stimulated with either saline (NaCl) or HDM for four days. In cell-free supernatants the cytokines IL-4, IL-5 and IL-13 (**a**-**c**) were measured. In a separated experiment mLN cells were incubated with PMA and ionomycin in the presence of Golgiplug, followed by intracellular staining of IL-4 (**d**) and intranuclear staining of GATA-3 (**e**). Data are depicted as means ± SEM (n = 6–8 per group). *#P* < 0.05, *##P* < 0.01, ***P < 0.001 for comparison between NaCl and HDM within control or anti-C5 mice

**Fig. 6:**
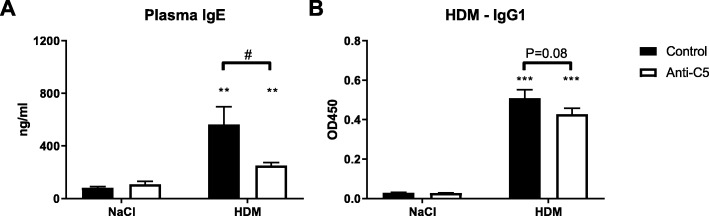
C5 inhibition reduces immunoglobulin release in plasma following HDM challenge. (**a**) IgE concentration and (**b**) absorbance (OD450) of HDM-specific IgG1 in plasma. Data are express as means ± SEM (n = 6–8 per group). *#P* < 0.05, **P < 0.01 and ***P < 0.001 for comparison between NaCl and HDM within control or anti-C5 mice

To further evaluate type 2 responses, we measured T_H_2 cytokines in BAL fluid and supernatants of mLNs re-stimulated with HDM. As expected, HDM challenge elicited enhanced IL-5 and IL-13 release in BAL fluid compared with saline controls (Fig. 4a, b). C5 inhibition did not alter the levels of these T_H_2 cytokines in BAL fluid. IL-4 remained below detection limit in BAL fluid, consistent with previous results from our group [24]. Re-stimulation of mLNs obtained from HDM challenged mice with HDM resulted in release of IL-4, IL-5 and IL-13 (Fig. 5a-c). C5 inhibition during the HDM challenge phase was associated with diminished IL-4 release by mLN upon re-exposure to HDM, while IL-5 and IL-13 release were not modified. The attenuated IL-4 release by mLN in C5 inhibited mice was accompanied by reduced intracellular IL-4 production by mLN derived CD4 T-cells (Fig. 5d) and a lower proportion of CD4^+^GATA-3^+^ T-cells in mLN (Fig. 5e). During allergic inflammation, IL-4 mediates the class switch recombination of IgM to IgE and IgG1 in B-cells [22]. Repeated HDM challenge led to elevated plasma IgE and HDM-specific IgG1 concentrations (Fig. 6a, b). C5 inhibition reduced total IgE and tended to lower HDM-specific IgG1 (*P* = 0.08). Together these data show that C5 inhibition during the HDM challenge phase attenuates type 2 responses without affecting ILC2s.

### C5 inhibition does not modify lung pathology following HDM challenge

**Fig. 7:**
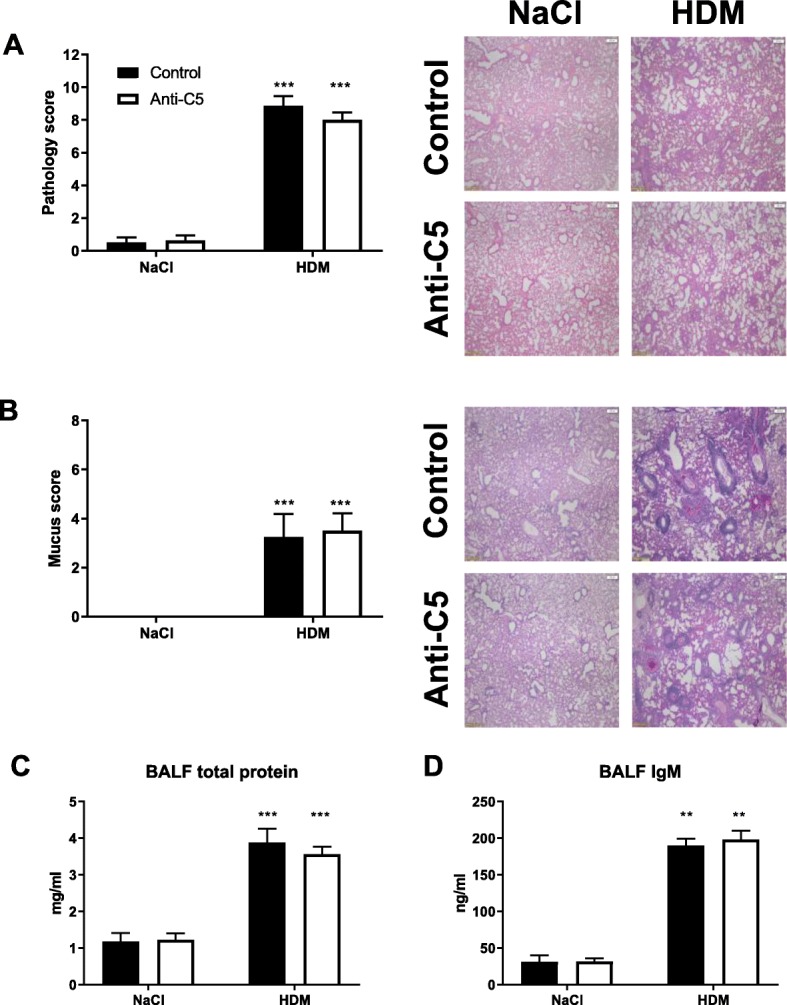
C5 inhibition does not alter lung pathology and vascular permeability following HDM challenge. (**a**) Total pathology score of hematoxylin and eosin (H&E) stained lung sections (× 4 magnification) from control (black bar) and BB5.1 (white bar) treated mice and (**b**) total mucus score of PAS-D stained lung sections(× 4 magnification) (n = 6–8 per group). Protein leakage was assessed using (**c**) total protein and (**d**) IgM in BALF (n = 6–8 per group). **P < 0.01 and ***P < 0.001 for comparison between NaCl and HDM within control or anti-C5 mice

This model of HDM induced allergic inflammation reproduces important features of asthma such as perivascular and interstitial inflammation, peribronchitis, endothelialitis and oedema, as well as mucus production [24] (Fig. 7a, b). Anti-C5 treated mice displayed HDM-induced lung inflammation to the same extent as control mice, as reflected by the semi-quantitative scoring system described in the Methods section. Likewise, HDM challenge evoked mucus production was unaltered in anti-C5 administered mice. Complement C5 has been implicated to play a role in vascular permeability [26, 27]. We measured total protein and IgM in BAL fluid as measures for vascular leak. C5 inhibition in HDM challenged mice did not attenuate allergen induced vascular leak (Fig. 7c, d).

### C5 inhibition reduces airway hypersensitivity as measured by PenH

**Fig. 8:**
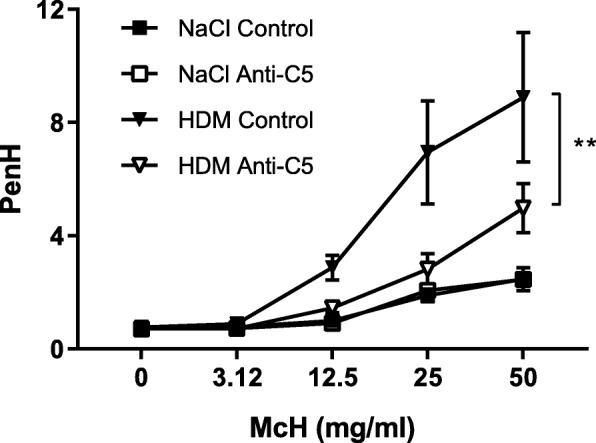
C5 inhibition attenuates AHR following HDM challenge. Airway hypersensitivity (AHR) expressed as enhanced pause (PenH) after dose-response to methacholine (Mch) exposure on day 19. Data are presented as means ± SEM (*n* = 6–8 per group). **P < 0.01

To obtain insight into the potential functional consequences of attenuated T_H_2 responses in C5 inhibited mice, we measured PenH as a marker for AHR (Fig. 8). Consistent with our previous studies [24] HDM sensitization and challenge elicited enhanced AHR in comparison to saline challenge. C5 inhibition significantly attenuated allergen induced AHR.

## Discussion

The anaphylatoxin C5a has been unveiled to play an important role in orchestrating the maladaptive T_H_2 immune response in murine asthma models [15, 16]. The mechanism by which C5a amplifies T_H_2 inflammation in an inflamed pulmonary environment is unclear. In the present study we demonstrate that C5 inhibition during the effector phase does not modulate the proportion of ILC2s but decreases T_H_2 cells in the lung and mLN, which is accompanied by reduced type 2 responses and an attenuated AHR.

Our HDM driven asthma model reproduces important features of allergic asthma such as pulmonary T_H_2 inflammation, AHR and airway mucus production [28]. In accordance with similar asthma models, HDM elicited an expansion of both ILCs and T_H_2 cells in the lungs [29, 30]. We here demonstrate for the first time that C5 inhibition during the effector phase does not modify the total ILC or ILC2 population in the lung, indicating that C5a signaling is not involved in ILC proliferation or differentiation. This finding is consistent with a study showing the lack of C5aR expression on ILC2s [31], rendering direct C5a signaling unlikely. Furthermore, specific deletion of C5aR on cells expressing the LysM promoter (such as neutrophils, macrophages and dendritic cells) in an OVA-model showed an equally strong increase in allergen evoked ILC2 cells compared to wild-type mice [32]. Our data indicate explicitly that C5 blockade during the effector phase hampers the differentiation of T_H_2 cells, resulting in reduced IL-4 production in mLN with unaltered IL-5 and IL-13 BALF levels. This discrepancy in T_H_2 cytokines has also been observed in previous studies in which C5aR was blocked during the effector phase in a murine asthma model [13, 18]. While the exact mechanism remains enigmatic, our data suggest that ILC2 derived IL-5 and IL-13 may have compensated a presumable lower IL-5 and IL-13 release from reduced T_H_2 cells numbers. Indeed, several studies reported enhanced IL-5 and IL-13 secretion by expanding ILC2s [33, 34], while ILC2s are generally considered either not to produce IL-4 [20, 22] or produce it in negligible amounts [35]. We found that C5 inhibition impeded IL-4 production in mLN which was associated with decreased plasma IgE and HDM-specific IgG1 responses. This finding is corroborated by a study which prevented C5 activation during the effector phase in an OVA-induced asthma model [19]. Conversely, abrogating C5 activation prior to sensitization yielded enhanced IgE production [14], emphasizing the time dependent dual character of C5a signaling during allergic inflammation. Remarkably, previous investigations demonstrated decreased eosinophil numbers without affecting IL-5 release in the airways following C5a signaling blockade [13, 18, 19]. The mechanism behind this disparity was not addressed. In our study, the absence of an effect on IL-5 and the eosinophil attractant CCL11 (data not shown) by anti-C5 treatment corresponded with a lack of differences in total leukocyte, especially eosinophil, influx in the airways. The unaltered airway leukocyte recruitment is in contrast to studies that likewise investigated C5a signaling in established asthma. Different allergens (i.e. OVA [19] or Aspergillus fumigatus [18]) used in these studies may have contributed. C5aR antagonists appeared to be potent to mitigate allergen induced leukocytes infiltration, including eosinophils, neutrophils and lymphocytes [13, 18] while blocking the anaphylatoxin C5a only limits neutrophil influx [19] or fails to affect leukocyte influx as shown by this study. Beside eosinophils, neutrophils express C5aR (CD88) on their cell membrane making direct C5a signaling possible [31]. C5a has been shown to be chemotactic for neutrophils and to promote release of neutrophil intracellular content [36]. Nonetheless, data from present study contradict an effect of C5a on neutrophil recruitment and neutrophil degranulation in HDM induced lung inflammation.

Consistent with earlier studies eliminating C5a/C5aR signaling during the effector phase [13, 18, 19], we observed an attenuated AHR in sensitized anti-C5 treated mice. Multiple factors may have contributed to this finding. First, a decreased total IgE concentration due to C5 inhibition could impair mast cell degranulation and consequently mast cell derived histamine induced bronchoconstriction. As C5a is an activator of mast cells, inhibited C5a activity could also influence AHR in a mast cell dependent manner [15]. Alternatively, C5a might aggravate AHR directly via activation of the C5aR on bronchial smooth muscle cells [37]. Our data argue against T_H_2 cytokine mediated attenuation of AHR, since IL-13 is essential for the regulation of AHR [38] and unaffected by C5 inhibition. IL-4 and IL-13 express high resemblance and transmit signals via shared functional receptor complexes (IL-4Rα/IL-13Rα1) [38]. Despite these similarities, a number of in vivo functional experiments have shown that IL-4 and IL-13 facilitate different features of allergic asthma. Specifically, IL-4 is regarded a regulator of T_H_2 cell proliferation and IgE synthesis [39–41] while IL-13 is thought to mediate AHR, mucus production, airway smooth muscle thickening and sub-epithelial fibrosis [42–45]. In accordance with the unaltered IL-13 concentration in BAL fluid, C5 inhibition did not modulate HDM induced mucus production. Although activation of C5 has been shown to enhance vascular permeability [46], C5 inhibition did not modulate vascular permeability in our HDM induced asthma model.

As result of C5 inhibition we observed reduced C5a levels which implies a simultaneously reduced formation of the membrane attack complex (MAC), since the assembly of the MAC occurs after cleavage of C5 into C5a and C5b [4]. While MAC drives numerous proinflammatory events and regulates cell signaling, its role in asthma has not been studied and the contribution of MAC in HDM-induced responses remains to be elucidated.

We used unrestrained whole body plethysmography (PenH) as measure for AHR. The use of this tool to measure airway resistance is under debate. However, the merit of this technique has been shown in various mouse models in which PenH results correlate well with results from invasive measurement techniques [47–49]. A close correlation between invasive measurements and whole body plethysmography has been validated in a comparable HDM induced asthma mouse model [48]. Moreover, AHR, measured by invasive methods, is enhanced following HDM challenge in models resembling ours [50, 51]. Nonetheless, interpretation of our Penh data without validation by invasive measurements is a limitation of this study.

## Conclusion

In conclusion, our data demonstrate that complement C5 activation during the challenge phase drives T_H_2 responses via T_H_2 cells but not ILC2s and aggravates allergen induced AHR. Further research is warranted to elucidate the underlying mechanisms of the C5a mediated effect on IL-4 production. Despite the promising beneficial effect of C5 inhibition on airway inflammation and AHR in murine asthma models, clinical investigations are needed to evaluate the therapeutic potential of C5 inhibition in asthma patients.

## Additional files

Additional file 1: **Figure S1.** Gating strategy for ILCs and ILC2s. Flow cytometry plots of lung tissue from (A) NaCl or (B) HDM challenged mice. From left to right; within the lymphocyte gate, single CD45 positive and viable cells expressing CD3^−^CD49b^−^Lin^−^CD90^+^ were defined as ILCs. Cells expressing ST2 positivity within the ILC population were defined as ILC2s. (PDF 206 kb)

Additional file 2: **Figure S2.** Gating strategy for CD4 T-cells and T_H_2 cells. Flow cytometry plots of lung tissue from (A) NaCl or (B) HDM challenged mice. From left to right; within the lymphocyte gate, single CD45 positive and viable cells expressing CD4 positivity were defined as CD4 T-cells. Cells expressing GATA-3 positivity within the CD4 population were defined as T_H_2 cells. (PDF 229 kb)

## Data Availability

The datasets used and/or analysed during the current study are available from the corresponding author on reasonable request.

## Abbreviations

AHR: Airway hyperresponsiveness
BAL: Bronchoalveolar lavage
C5aR: C5a receptor
DC: Dendritic cells
H&E: Hematoxylin and Eosin
HDM: House dust mite
ILC: Innate lymphoid cell
ILC2: Proliferation or group 2 ILC
mAb: Murine antibody
mLN: Mediastinal lymph nodes
PenH: Enhanced pause
T_H_2: T-helper type 2
