# Supplementary figures and images for: Complement factor C5 inhibition reduces type 2 responses without affecting group 2 innate lymphoid cells in a house dust mite induced murine asthma model
Source: Respir Res. 2019 Jul 24;20:165. doi: 10.1186/s12931-019-1136-5 (PMC6657208; doi:10.1186/s12931-019-1136-5)

Figure S1

A

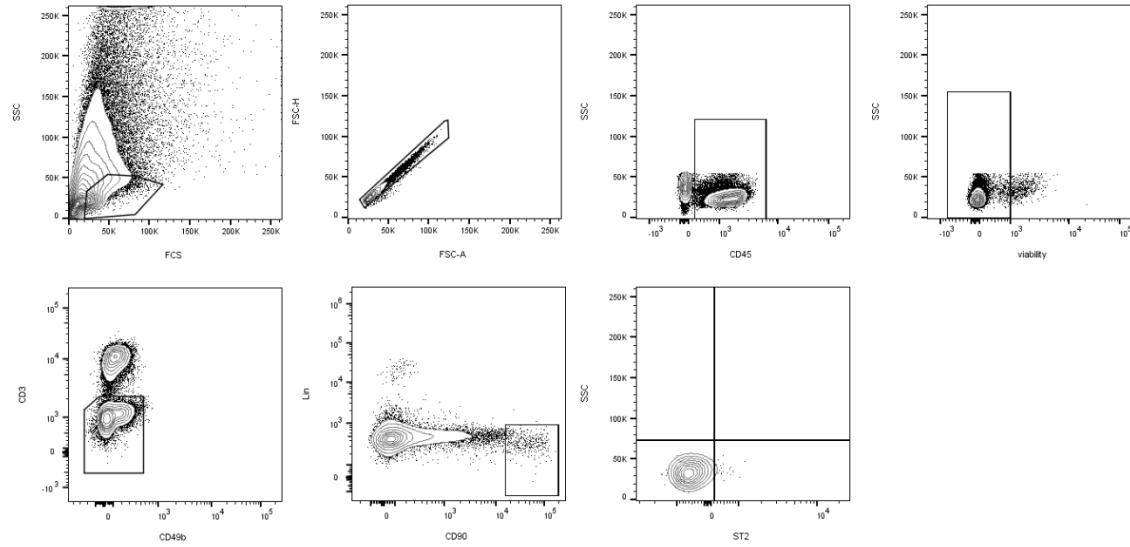

B

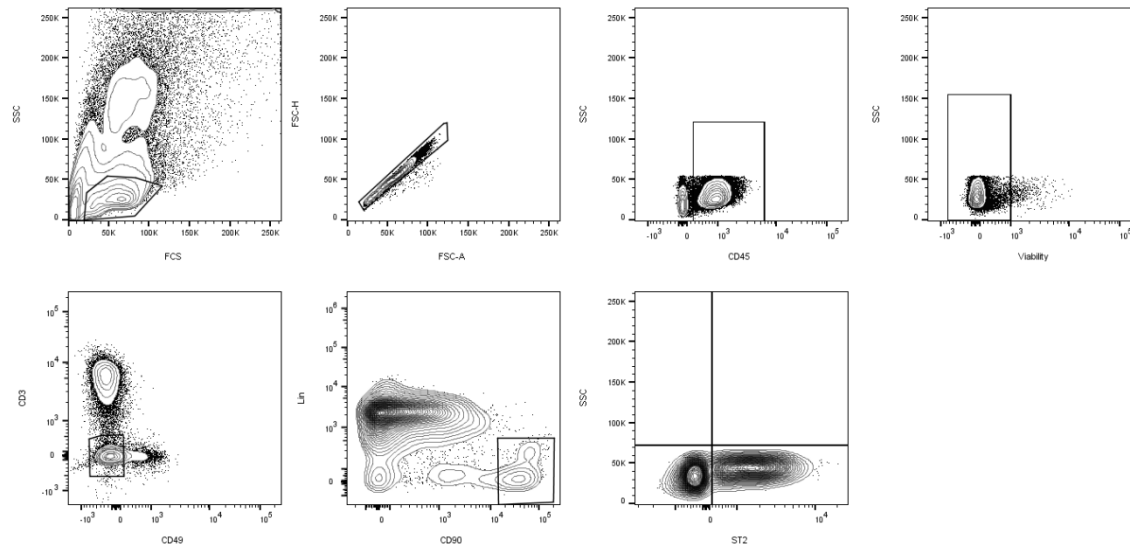

Supplement: Supplementary file 1 — Figure S1. Gating strategy for ILCs and ILC2s. Flow cytometry plots of lung tissue from (A) NaCl or (B) HDM challenged mice. From left to right; within the lymphocyte gate, single CD45 positive and viable cells expressing CD3−CD49b−Lin−CD90+ were defined as ILCs. Cells expressing ST2 positivity within the ILC population were defined as ILC2s. (PDF 206 kb) [file 12931_2019_1136_MOESM1_ESM.pdf]

Figure S2

A

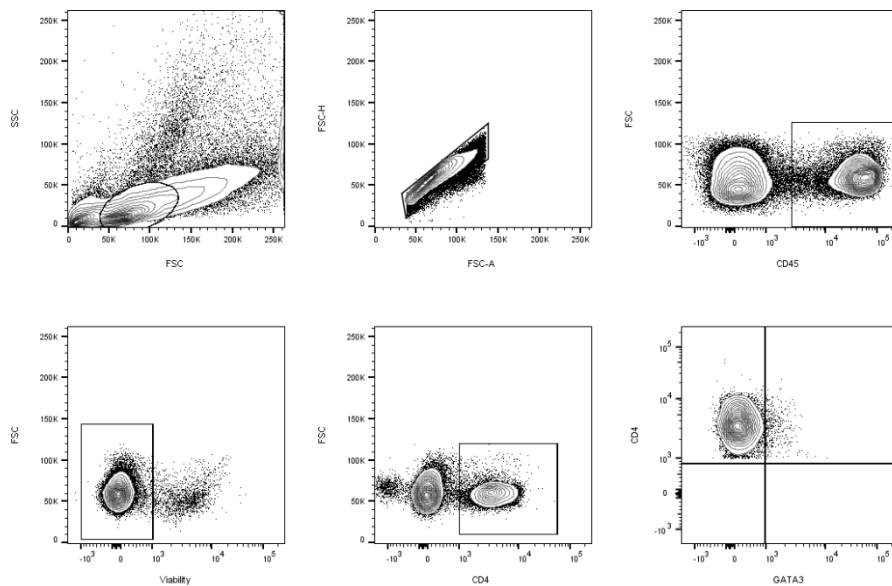

B

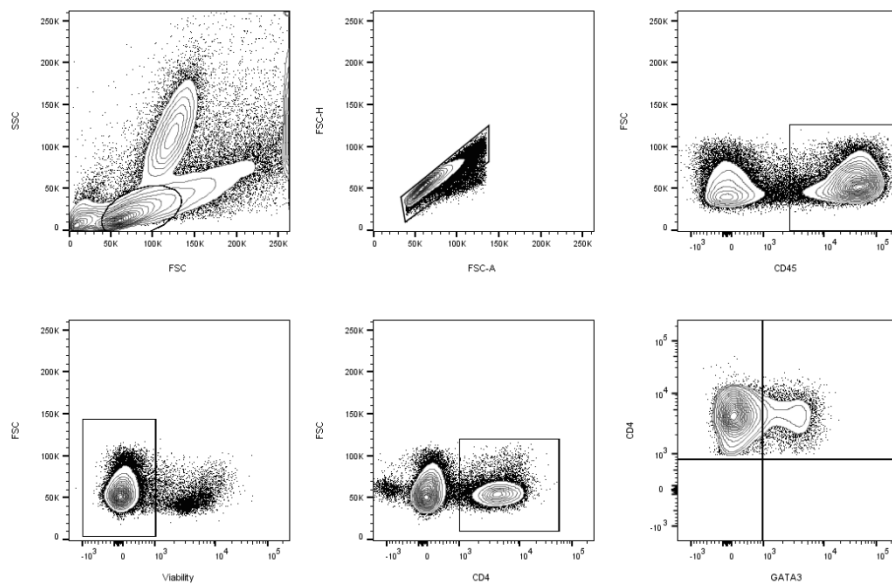

Supplement: Supplementary file 2 — Figure S2. Gating strategy for CD4 T-cells and TH2 cells. Flow cytometry plots of lung tissue from (A) NaCl or (B) HDM challenged mice. From left to right; within the lymphocyte gate, single CD45 positive and viable cells expressing CD4 positivity were defined as CD4 T-cells. Cells expressing GATA-3 positivity within the CD4 population were defined as TH2 cells. (PDF 229 kb) [file 12931_2019_1136_MOESM2_ESM.pdf]
